# Association Between Caregiver Burden and Treatment Adherence in Japanese Children With Allergic Rhinitis Receiving Sublingual Immunotherapy

**DOI:** 10.7759/cureus.74779

**Published:** 2024-11-29

**Authors:** Hikaru Matsuoka, Sadayuki Nagai, Takayasu Kanatani, Nao Kouho, Shintaro Inoue, Tsuneaki Kenzaka

**Affiliations:** 1 Department of Pediatrics, Hyogo Prefectural Tamba Medical Center, Tamba, JPN; 2 Department of Internal Medicine, Hyogo Prefectural Tamba Medical Center, Tamba, JPN; 3 Division of Community Medicine and Career Development, Kobe University Graduate School of Medicine, Kobe, JPN

**Keywords:** adherence, allergic rhinitis, caregiver burden, caregiver satisfaction, children, sublingual immunotherapy

## Abstract

Background: Sublingual immunotherapy (SLIT) leads to the long-term remission of allergic rhinitis and requires long-term daily adherence. There are limited studies on the treatment burden or satisfaction of SLIT among caregivers of children treated using SLIT. We aimed to evaluate the association between the treatment burden and satisfaction for pediatric allergic rhinitis caregivers and the clinical factors of their children's SLIT.

Methods: We conducted a questionnaire survey with 115 caregivers of SLIT-treated children from May to August 2023 and retrospectively reviewed their children`s medical records. A single caregiver had at least one child, all undergoing SLIT at the time of the survey. Of the total 139 SLIT-treated children, 78 children over eight years of age were interviewed regarding adherence to SLIT and subjective symptoms of allergic rhinitis. We performed a comparative analysis of caregivers' impressions of SLIT and their children's clinical factors regarding SLIT.

Results: A runny nose was the most common symptom experienced by children before the start of SLIT. After the children started SLIT, 74 (64.3%) of the 115 caregivers felt satisfied and 30 (26.1%) felt burdened by SLIT. The most frequent reasons for being satisfied and burdened by SLIT were because it improved or relieved allergic rhinitis and the preparation and encouragement for children to take SLIT tablets, respectively. Of the 115 caregivers, 96 (83.4%) recommended SLIT to others. A comparative analysis showed that caregivers had already felt burdened from the first one to six months of SLIT initiation and the proportion of caregivers who answered that they felt burdened by SLIT was significantly lower for children with appropriate adherence than for those with inappropriate adherence (11.1% vs. 30.9%,*P = 0.034*).

Conclusions: Most caregivers of SLIT-treated children reported higher treatment satisfaction than treatment burden. Caregiver burden emerged shortly after the start of SLIT and was also associated with lower adherence in children. This study emphasized the need for strategies to support caregivers and optimize adherence in the early stages of SLIT.

## Introduction

In Japan, the proportion of allergic rhinitis among young individuals has recently increased. According to a national epidemiological survey in 2019, cedar pollinosis was found in 3.8% of children aged zero to four years and 30.1% of children aged five to nine years, and perennial allergic rhinitis was found in 5.1% of children aged zero to four years and 20.9% of children aged five to nine years, both with high prevalence rates [1]. Children with allergic rhinitis experience poor concentration, test scores, and motor skills [2,3], while caregivers of children with allergic rhinitis stated that their children had poor mental health and well-being [4]. There has long been a desire for curative therapy for allergic rhinitis that improves the quality of life of affected children and their caregivers. However, children with allergic rhinitis cannot be expected to acquire immunity or be cured during its natural course and require long-term management [5].

Allergen immunotherapy, the only curative therapy that can modify the natural history, is attracting attention. Sublingual immunotherapy (SLIT) is one of the safest forms of allergen immunotherapy; in 1986, Scadding et al. [6] reported the first SLIT using a small amount of mite extract. Magrogna et al. [7] prospectively evaluated the efficacy of SLIT with mite extract in adult patients treated for three, four, and five years and reported that long-term efficacy was achieved not only during treatment but also seven to eight years after treatment was discontinued. In addition, Okubo et al. [8] conducted a randomized controlled trial (RCT) in Japan in which adult patients with cedar pollinosis were assigned to SLIT with cedar pollen extract or placebo and reported high efficacy.

In several clinical trials in Japan, Cedarcure® (Japanese cedar pollen SLIT tablet) and Miticure® (mite SLIT tablet) have shown efficacy and safety, respectively [9-12]. These two tablets were approved for insurance coverage for pediatric allergic rhinitis in Japan in 2018 and were actively used at Hyogo Prefectural Tamba Medical Center in 2019. The appropriate use of Cedarcure® and Miticure® is a once-daily dose and long-term continuation for three to five years, and SLIT requires adherence. While it is important for caregivers to help their children take medication to ensure adherence, there is a concern that such help can be a substantial treatment burden among caregivers.

Therefore, we conducted a survey on the caregivers of children treated with SLIT because there are limited reports on treatment satisfaction and burden among caregivers of children with allergic rhinitis treated with SLIT. The aim of this survey was to evaluate the association between the treatment burden and satisfaction of SLIT for Japanese pediatric allergic rhinitis caregivers and the clinical factors of their children's SLIT.

## Materials and methods

Study design

We conducted a study at the Department of Pediatrics, Hyogo Prefectural Tamba Medical Center, Tamba, Japan, from May to August 2023, using an anonymous questionnaire, on caregivers of children aged five to 18 years who were undergoing SLIT at our hospital. The following clinical data were retrospectively collected from their children’s medical records: sex, age at SLIT initiation, duration of SLIT, type of SLIT, comorbid allergic diseases, and allergy-related serum immunological tests. All of the SLIT-treated children were included in this study because there is no such data loss.

First, we distributed the questionnaire to the caregivers during regular outpatient visits and asked them to complete it. Second, we received the completed questionnaire face to face and briefly interviewed the caregivers. During the study period, each caregiver responded to the questionnaire only once. When the same parent visited with several children treated using SLIT, instead of obtaining answers for each child, one answer was shared for all children. Third, we also interviewed only the SLIT-treated children aged ≥8 years regarding their adherence to SLIT and subjective symptoms of allergic rhinitis. The SLIT-treated children <8 years were excluded from our interviews because we found that as the age of the children interviewed decreased, their answers became less reliable. Finally, we categorized the children's clinical factors regarding SLIT (duration of SLIT, type of SLIT, age at SLIT initiation, with/without siblings undergoing SLIT, SLIT adherence, and subjective symptoms) into groups and calculated the proportion of caregivers' impressions of SLIT for each group. We compared the proportion of caregivers' impressions between these groups.

Approval was obtained from the Ethics Committee of the Hyogo Prefectural Tamba Medical Center (ethics committee approval number: TAN-I 1037), and informed consent was obtained from all participants.

Summary of the questionnaire

The summary of the questionnaire (see Appendix) is as follows: (1) date of response, age and gender of the child, relationship with the child, and details of SLIT (Nos. 1-5); (2) caregivers’ impressions of treatment before starting SLIT (Nos. 6-8); and (3) caregivers’ impressions of treatment after starting SLIT (Nos. 9, 10). Considering the time burden among caregivers, 10 questions were selected so that all questions could be answered within five minutes. 

Children with allergic rhinitis treated using SLIT

All children treated using SLIT fulfilled the following criteria: (i) allergy-related serum immunological tests (total IgE level, Japanese cedar pollen (JCP)-specific IgE antibody, and *Dermatophagoides pteronyssinus* (Der)-specific IgE antibody) were performed at our hospital or at another clinic before SLIT initiation, (ii) SLIT initiation was performed at our hospital, and (iii) SLIT was continued at our hospital. Serum JCP- and Der-specific IgE antibodies were measured using either the single allergen test (ImmunoCAP® assays, Thermo Fisher Diagnostics Co., Ltd., Tokyo, Japan) or the multiple allergen test (View39® assays, Thermo Fisher Diagnostics Co., Ltd., Tokyo, Japan). The ImmunoCAP® and View39® assays are interchangeable, both assays have high diagnostic concordance rates, and both are commonly used to measure specific IgE antibodies in patients with allergic rhinitis.

SLIT tablets

The tablets used for SLIT were Cedarcure® and Miticure® (Torii Pharmaceutical Co., Ltd., Tokyo, Japan). We reviewed the contraindications and precautions on the package inserts and then administered these tablets to patients with allergic rhinitis for a one-week increased dose (Cedarcure®: 2000 Japanese allergy units (JAU), Miticure®: 3300 JAU), followed by a maintenance dose (Cedarcure®: 5000 JAU, Miticure®: 10000 JAU). The medication was administered once daily before sleep. Supportive care medications, such as antihistamines and steroid nasal sprays, were administered at the discretion of the attending physician.

Definitions

JCP allergic rhinitis was defined as having rhinitis symptoms limited to the cedar pollen dispersal period (February to April) in Japan and JCP-specific IgE antibody class ≥2 (both ImmunoCAP® assays and View39® assays). Perennial allergic rhinitis was defined as having rhinitis symptoms not only from February to April but also throughout the year and Der-specific IgE antibody class ≥2. Dual SLIT was defined as SLIT with both Cedarcure® and Miticure®, and their duration of and age at SLIT initiation were calculated based on the time of introduction of the first sublingual tablet. Inappropriate adherence was defined as self-administration of SLIT tablets for six days or less per week. Appropriate adherence was defined as self-administration of SLIT tablets every day or almost every day.

Statistical analysis

Statistical analyses were performed using Microsoft Office Excel 2019 (Microsoft Corporation, USA). Continuous variables were expressed as medians (interquartile range, IQR). Categorical variables were expressed as percentages and compared using Fisher’s exact test. Descriptive statistics including percentages, medians, and IQRs were calculated. We analyzed open-ended answers in “question 9” according to the “after-cording method,” with generative artificial intelligence ChatGPT 3.5 (Open AI, Inc., San Francisco, USA). Adjustments for multiple comparisons were not planned for this study because it was an exploratory study. All p-values were set as significant, with a two-tailed test of <0.05.

## Results

Between May and August 2023, 115 caregivers accompanying 139 children treated using SLIT were asked to complete a questionnaire, and all 115 responded. One (0.8%) of the 115 caregivers responded twice during the study period; therefore, only the first response was included in this analysis. Of the 115 caregivers, 96 mothers (83.5%), 14 fathers (12.2%), and five grandmothers (4.3%) responded to the questionnaire. All five grandmothers were the primary caregivers because their grandchildren's parents were divorced. The backgrounds of the 139 SLIT-treated children with allergic rhinitis are shown in Table 1. Ninety-six (69.0%) of the 139 children were boys, the median age at SLIT initiation was 7.0 years (IQR, 5.5-9.5 years), and the median SLIT duration was 21.0 months (IQR, 16.5-23.2 months). Sixty-one (43.8%) were in the Miticure® alone group, 17 (12.4%) were in the Cedarcure® alone group, and 61 (43.8%) were in the dual SLIT group. Comorbid allergic diseases included bronchial asthma in 60 patients (43.1%), atopic dermatitis in 29 patients (20.8%), and food allergy in 25 patients (17.9%).

**Table 1:** Background of the children with allergic rhinitis treated using SLIT SLIT: sublingual immunotherapy; Der: *Dermatophagoides pteronyssinus*; JCP, Japanese cedar pollen Data are presented as number (percentage) or median (interquartile range).

Backgrounds	Children (n = 139)
Demographic	
Male, n (%)	96 (69.0%)
Age at the start of SLIT, (years)	7.0 (5.5-9.5)
Duration of SLIT, (months)	21.0 (16.5-23.2)
The type of SLIT, n (%)	
Miticure^®^	61 (43.8%)
Cedarcure^®^	17 (12.4%)
Dual SLIT	61 (43.8%)
Serum immunological tests (before SLIT)	
Total IgE, (IU/ml)	534 (205-1237)
Der-specific IgE	
Class (2/3/4/5/6) (ImmunoCAP^® ^assays), (n)	9/ 16/ 16/ 17/ 34
Class (2/3/4/5/6) (View39^®^ assays), (n)	0/ 5/ 10/ 8/ 11
JCP-specific IgE	
Class (2/3/4/5/6) (ImmunoCAP^® ^assays), (n)	8/ 21/ 16/ 10/ 17
Class (2/3/4/5/6) (View39^®^ assays), (n)	3/ 8/ 8/ 2/ 5
Comorbid allergic diseases	
Bronchial asthma, n (%)	60 (43.1%)
Atopic dermatitis, n (%)	29 (20.8%)
Food allergy, n (%)	25 (17.9%)

Caregivers’ impressions of treatment before starting SLIT

**Figure 1:**
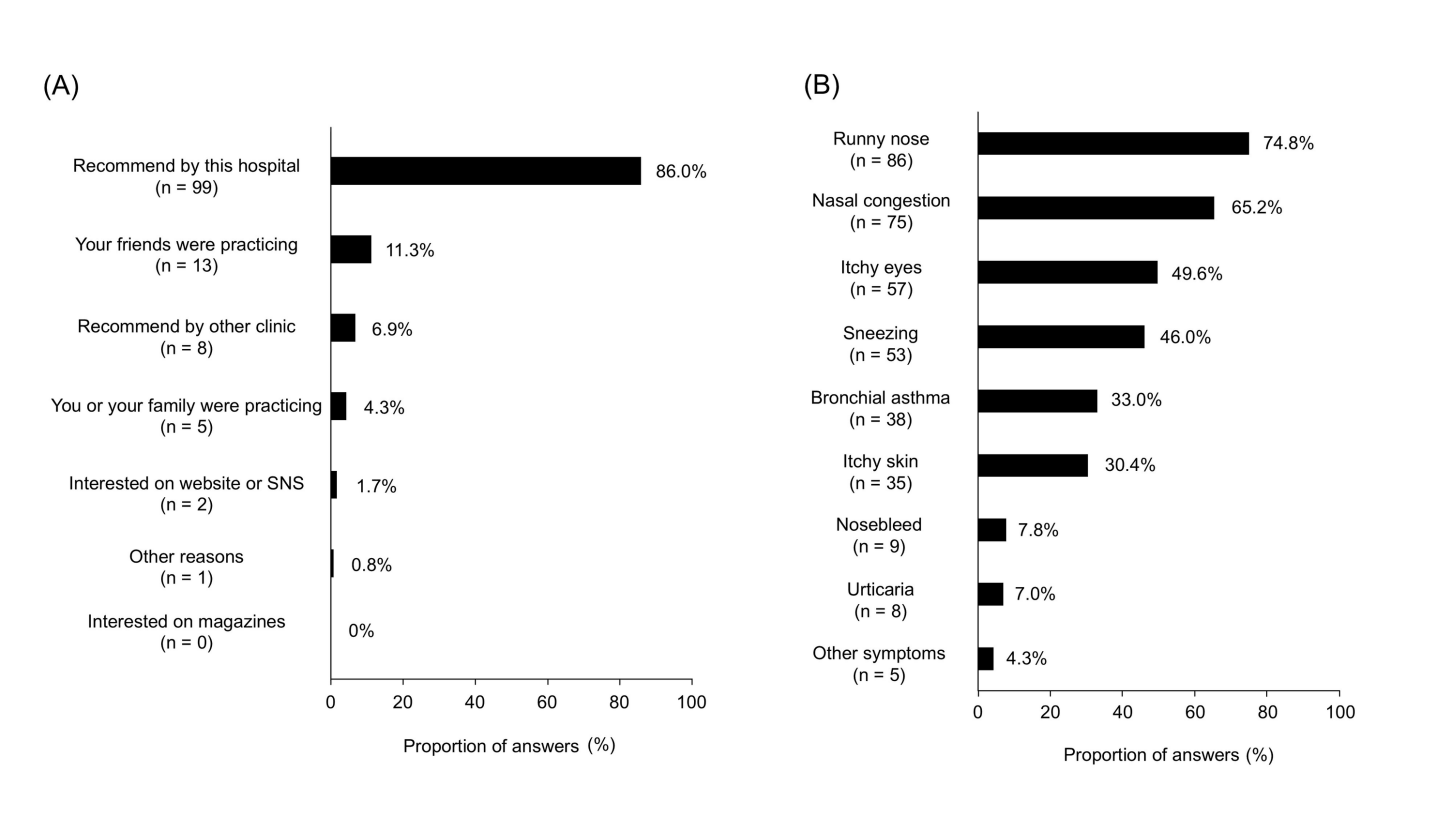
Caregivers’ impressions of treatment before starting SLIT (A) The reasons why children started SLIT. (B) The children’s allergic symptoms before starting SLIT. SLIT, sublingual immunotherapy.

For question 6, “How and why did you start sublingual immunotherapy?”, of the 115 caregivers, 99 caregivers (86.0%) responded “recommended by this hospital,” 13 (11.3%) responded “your friends were practicing,” and eight (6.9%) responded “recommended by other clinics” (Figure 1A). For question 7, “What allergic symptoms prompted your child to start sublingual immunotherapy?”, 86 caregivers (74.8%) responded “runny nose,” 75 (65.2%) responded “nasal congestion,” and 57 (49.6%) responded “itchy eyes” (Figure 1B).

For question 8, “What did you expect from the treatment before starting sublingual immunotherapy?”, 82 caregivers (71.4%) answered “both symptoms and anti-allergy medication would decrease, making daily life easier,” 30 (26.1%) answered “complete disappearance of symptoms,” one (0.8%) answered “not much hope, just a little relief of symptoms,” and two (1.7%) answered “no answer.”

Caregivers’ impressions of treatment after starting SLIT

For question 9, “Please give us your honest opinion about sublingual immunotherapy currently being offered. First, please choose the most appropriate answer in parentheses regarding your impression of the treatment,” 74 caregivers (64.3%) answered that they felt satisfied with the treatment, 30 (26.1%) answered that they felt burdened, seven (6.1%) were neutral, and four (3.5%) gave no answer. Then, the caregivers provided an open-ended answer as to why they judged the treatment to be "a satisfaction" or "a burden.” Among the 115 caregivers, 103 (89.6%) freely wrote in the “I feel satisfied with the treatment” column (Table 2). Of these 103 caregivers, 57 caregivers (55.3%) responded that “allergic rhinitis was relieved or improved," followed by 15 (14.6%) each for “cedar pollinosis was relieved or improved” and “bronchial asthma was relieved or improved” in the symptom category. Moreover, the most common reason for the treatment category was “taking the SLIT tablet became a habit” for 13 caregivers (12.6%), and the most common reason for the lifestyle category was "quality of exercise and study got better" for four caregivers (3.9%).

**Table 2:** Open-ended answers from caregivers who feel satisfied with SLIT SLIT: sublingual immunotherapy Data are presented as numbers (percentage). † Other allergic diseases are food allergy (n = 2) and atopic dermatitis (n = 1).

Answer by category	Caregivers (n = 103)
Symptom	
Allergic rhinitis (except cedar pollen) relieved or improved	57 (55.3%)
Cedar pollinosis relieved or improved	15 (14.6%)
Bronchial asthma relieved or improved	15 (14.6%)
Other allergic diseases† relieved or improved	3 (2.9%)
Medication	
Taking the SLIT tablet became a habit	13 (12.6%)
Expectation for future therapeutic benefits of SLIT	8 (7.8%)
Easy to take orally disintegrating tablets	3 (2.9%)
Use of antihistamines decreased	3 (2.9%)
Feel secure in receiving regular checkups	1 (1.0 %)
Lifestyle	
Quality of exercise or study got better	4 (3.9%)
Quality of sleep got better	2 (1.9%)
Bathing more regularly	1 (1.0%)
Increased interest in allergic diseases	1 (1.0%)

Next, 48 (41.7%) of the 115 caregivers provided an open-ended answer to the “I feel burdened by the treatment” column (Table 3). Of these 48 caregivers, the most frequent reason given was “encourages and prepares to take the SLIT tablet” by 21 caregivers (43.8%), followed by “feeling the burden in adherence to the SLIT tablet” by 13 caregivers (27.1%) and “side effects localized to oral cavity” by eight caregivers (16.7%). For question 10, “Would you recommend sublingual immunotherapy to your friends or family members?”, of the 115 caregivers, 96 (83.4%) answered “yes,” nine (7.8%) answered “no,” four (3.5%) answered “neutral,” and six (5.4%) gave no response.

**Table 3:** Open-ended answers from caregivers who feel burdened by SLIT SLIT: sublingual immunotherapy Data are presented as numbers (percentage).

Answer by category	Caregivers (n = 48)
Symptom	
Side effects localized to oral cavity	8 (16.7%)
Not feeling relief or improvement of symptoms of allergic rhinitis	3 (6.3%)
Medication	
Encourages and prepares to take the SLIT tablet	21 (43.8%)
Feeling burden in adherence to the SLIT tablet	13 (27.1%)
Difficulty in taking the SLIT tablet before sleep	3 (6.3%)
High cost of the SLIT tablet	2 (4.2%)
Difficulty in taking the SLIT tablet following the package insert	2 (4.2%)
Lifestyle	
Long waiting time for outpatient visits	1 (2.1%)
Increased nocturia with water intake when taking the SLIT tablet	1 (2.1%)

Comparison between caregivers' impressions and their children's clinical factors regarding SLIT

**Figure 2:**
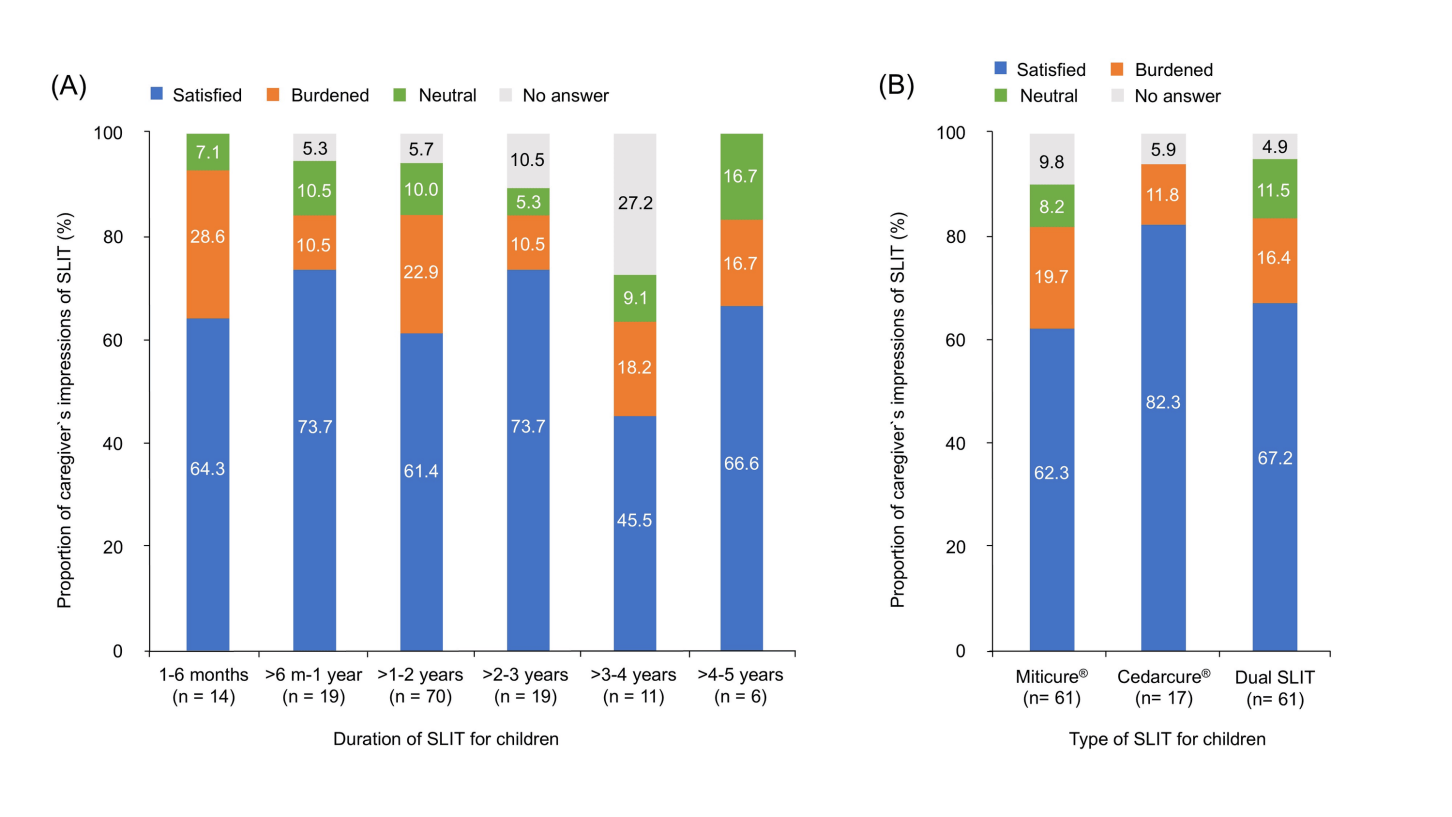
Comparison between caregivers’ impressions of SLIT and SLIT condition of their children (A) Proportion of caregivers’ impressions of SLIT (*Y-axis*) for each child’s SLIT duration, expressed as a stacked bar chart (satisfied (*blue*), burdened (*orange*), neutral (*green*), no answer (*gray*)). (B) For each type of SLIT, the proportion of caregivers’ impressions of SLIT (*Y-axis*) was expressed (satisfied (*blue*), burdened (*orange*), neutral (*green*), no answer (*gray*)). SLIT, sublingual immunotherapy.

In a comparison of the caregivers' impressions of their children’s duration of SLIT, the proportion of caregivers who felt satisfied with SLIT was over 60% for all groups, except for the group of children with a SLIT duration of three to four years. The proportion of caregivers who felt burdened by SLIT was the highest in the group of children with a SLIT duration of one to six months (one to six months: 28.6%; >6 months to one year: 10.5%; >1-2 years: 22.9%; >2-3 years: 10.5%; >3-4 years: 18.2%; >4-5 years: 16.7%; Figure 2A). The proportion of caregivers who felt satisfied with SLIT when the type of SLIT for children was Cedarcure® alone was higher than the proportion when the type of SLIT for children was Miticure® alone or dual SLIT (Miticure®: 62.3%; Cedarcure®: 82.3%; dual SLIT: 67.2%; Figure 2B).

**Figure 3:**
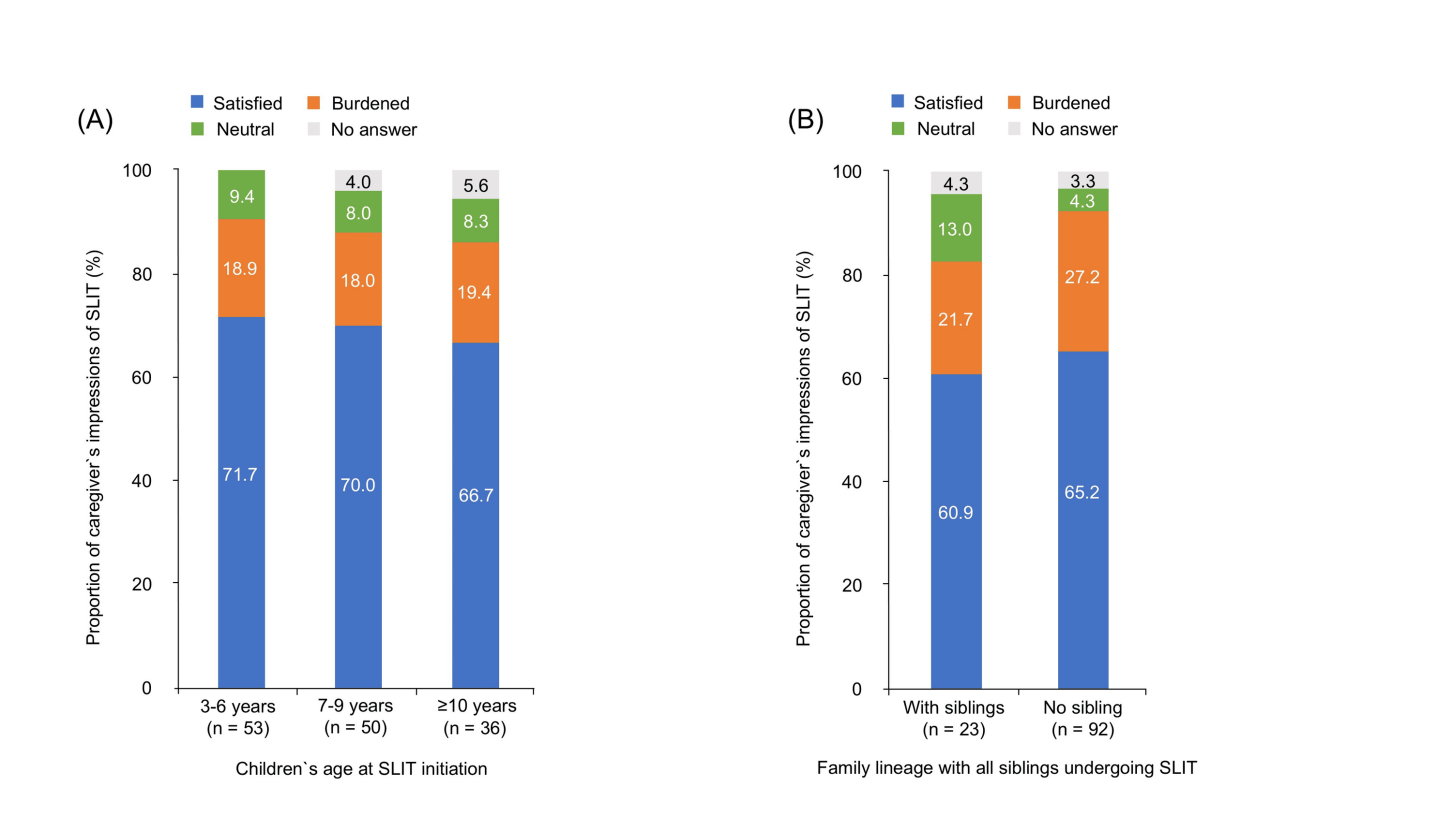
Comparison between caregivers’ impressions of SLIT and SLIT condition of their children (A) Proportion of caregivers’ impressions of SLIT (*Y-axis*) for children’s age at SLIT initiation, expressed as a stacked bar chart (satisfied (*blue*), burdened (*orange*), neutral (*green*), no answer (*gray*)). (B) Proportion of caregivers’ impressions of SLIT (*Y-axis*) was expressed depending on the presence or absence of SLIT siblings in the same family (satisfied (*blue*), burdened (*orange*), neutral (*green*), no answer (*gray*)). SLIT, sublingual immunotherapy.

The proportion of caregivers’ impressions was almost the same for all ages of children (Figure 3A). There were 23 family lineages, with all siblings being treated using SLIT. There was no significant difference in the proportion of caregivers who answered that they felt satisfied with the treatment (with siblings vs. without = 60.9% vs. 65.2%,* P = 0.697*) or burdened (with siblings vs. without = 21.7% vs. 27.2%,* P = 0.595*) (Figure 3B).

**Figure 4:**
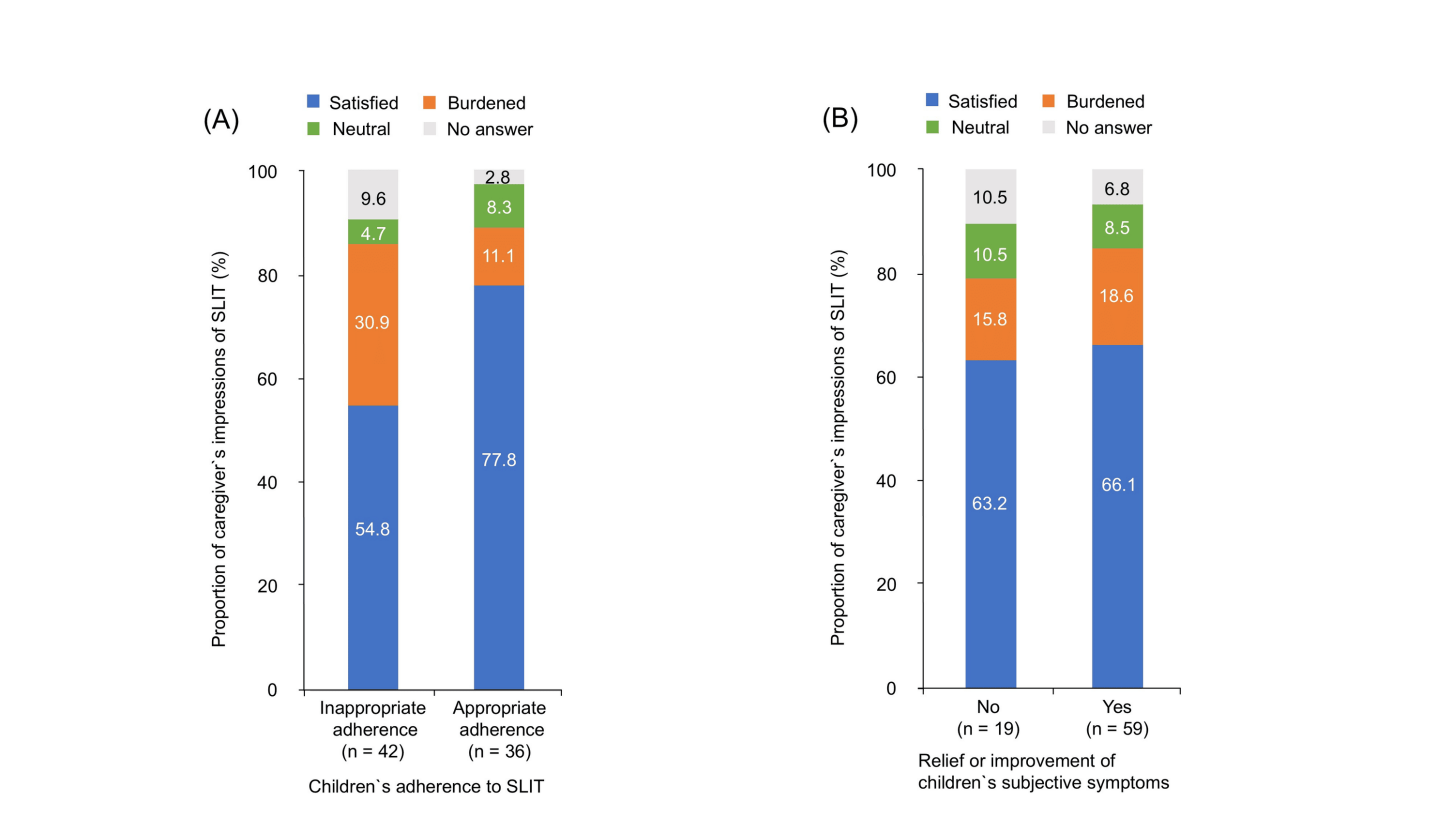
Comparison between caregivers’ impressions of SLIT and SLIT condition of their children (A) Proportion of caregivers’ impressions of SLIT (*Y-axis*) for each child’s adherence to SLIT, expressed as a stacked bar chart (satisfied (*blue*), burdened (*orange*), neutral (*green*), no answer (*gray*)). (B) Proportion of caregivers’ impressions of SLIT (*Y-axis*) was expressed depending on with or without each child`s subjective symptoms of allergic rhinitis (satisfied (*blue*), burdened (*orange*), neutral (*green*), no answer (*gray*)). SLIT, sublingual immunotherapy.

Of the 139 children, 78 (56.1%) were over eight years of age. We interviewed the 78 children regarding their adherence to SLIT and subjective symptoms of allergic rhinitis. Among the 78 children, 42 (53.8%) had inappropriate adherence, while the remaining 36 (46.2%) had appropriate adherence. The proportion of caregivers who answered that they felt burdened by SLIT was significantly lower for children with appropriate adherence than for those with inappropriate adherence (11.1% vs. 30.9%, *P = 0.034*) (Figure 4A). Fifty-nine (75.6%) of the 78 children reported that their symptoms of allergic rhinitis had relieved or improved, while 19 (24.4%) reported that their symptoms had not. There was no significant difference in the proportion of caregivers' impressions between relief or improvement of children`s symptoms of allergic rhinitis and without (Figure 4B).

## Discussion

This cross-sectional study using a questionnaire showed that the proportion of caregivers who felt satisfied with SLIT was higher than that of caregivers who felt burdened by the treatment (26.1% (30/115) vs. 64.3% (74/115)). The most frequent reason for satisfaction with SLIT was improvement or relief of allergic rhinitis in children. Just as 112 (97.6%) of the 115 caregivers expected their children's allergic symptoms to improve before starting SLIT, 96 (83.4%) of the 115 caregivers said they would recommend SLIT to others. Given the nature of children, it is difficult to objectively evaluate the therapeutic efficacy of SLIT in pediatric allergic rhinitis. Nasal endoscopic evaluations, such as those performed by otolaryngologists, are not easy for pediatricians. Ishiguro et al. [13] objectively evaluated the efficacy of SLIT in children using a visual analog scale. In this study, we were not able to objectively evaluate the severity and level of clinical symptoms of allergic rhinitis by nasal endoscopy or a visual analog scale. However, pediatricians frequently encounter situations in which we must refer to caregivers’ comments to assess a child's adherence to medication and daily symptom changes. As a result, we found that caregivers’ comments, in addition to objective assessments by physicians, may be useful as a tool for evaluating the effectiveness of pediatric SLIT.

SLIT requires long-term daily administration. However, it is extremely difficult for children to maintain adherence [14]. In a survey of adherence in 150 Italian children with SLIT under six years of age, 80.1% of the three-to-four-year-old group, 32.2% of the four-to-five-year-old group, and 26.8% of the five-to-six-year-old group had poor adherence, indicating that the least adherence was achieved in the youngest age group [15]. In an RCT of 300 Italian pediatric SLIT cases, aged six to 16 years, examining the relationship between adherence and frequency of hospital visits, the proportion of children discontinuing SLIT substantially increased as the frequency of hospital visits decreased from four times per year to twice per year to once per year [16]. Medication support by caregivers is essential for achieving adherence to SLIT in pediatric patients. This study revealed that the need to encourage and prepare their children to take the SLIT tablets was the most frequent reason for caregivers to feel the burden of SLIT, and approximately 30% of caregivers of children whose SLIT duration ranged from one to six months, the period shortly after the start of SLIT, already felt burdened. Moreover, we found a significant association between caregivers' treatment burden and their children's inappropriate adherence. Physicians in charge of pediatric SLIT need to be encouraged by the hard work of caregivers who struggle to maintain their children's adherence from the beginning of SLIT to make pediatric SLIT a success.

Another key to successful pediatric SLIT is good management of side effects, which are mainly lesions localized to the oral cavity (e.g., oral pruritus and swelling of the lips, tongue, and pharynx). These side effects are characterized by a high incidence during the first phase of SLIT (e.g., the dose-increasing phase; Cedarcure® 2000 JAU, Miticure® 3300 JAU), duration 10 to 60 minutes, persistence for two weeks after SLIT initiation, and spontaneous disappearance. In clinical trials, 4-7% of patients drop out because of side effects [17-21]. This study showed that eight (16.7%) of the 48 comments from caregivers who felt burdened by SLIT were related to side effects, all of which were oral pruritus. Yuta et al. [22] reported that a change to spitting SLIT tablets instead of swallowing them should be tried for relief and prevention of oral pruritus. In our own approach, we instructed our pediatric patients to administer antihistamines a few minutes before the SLIT tablet during the first few months of the maintenance phase, as well as during the SLIT dose increase phase. This approach has proven useful for reducing side effects. Prevention of side effects in children is very important, not only because it reduces the treatment burden among caregivers, but it also avoids self-discontinuation of the children's treatment.

The international guidelines on pediatric bronchial asthma published by the European Academy of Allergy and Clinical Immunology in 2019 and the Global Initiative for Asthma in 2021 state that SLIT is recommended as an additional treatment for childhood bronchial asthma cases with mild to moderate disease and is expected to reduce symptoms of bronchial asthma and decrease the use of inhaled medications [23,24]. The 2023 Japanese guideline for pediatric bronchial asthma suggests that mite-allergen-specific immunotherapy should be a standard treatment for pediatric bronchial asthma with mite allergy [25]. In this study, 15 (14.6%) of the 103 caregivers who felt satisfied with SLIT responded that their children’s bronchial asthma was reduced or relieved. The number of children cared for by these 15 caregivers was 15, who received the mite SLIT tablet (Miticure® alone: n = 9, dual SLIT: n = 6) with a median SLIT duration of 18 months (IQR: 15-35 months), suggesting that at least one year of SLIT duration is necessary for caregivers to feel a therapeutic effect of mite SLIT on their children's bronchial asthma. However, this study lacked clinical information on bronchial asthma management (bronchial asthma subjective symptom scores, drug dosage, and airway sensitivity test results) and could not be used to assess the effectiveness of SLIT for bronchial asthma. 

Limitation

This study has some limitations. First, this study is a single-center trial. The climate in Japan varies greatly by region. The prevalence of allergic rhinitis is easily influenced by climate, and the prevalence of allergic rhinitis varies among regions in Japan. A bias in patient selection may have occurred in this single-center study. This risk is limited by the selection of children with allergic rhinitis in a wide age range of five to 18 years. The strength of this study is that the questionnaire survey is conducted face to face, rather than web-based. Face-to-face questionnaires are generally administered in a method that allows for sufficient personal interaction, making this study reliable with a very high survey collection rate (100% (115/115)) and response rate to open-ended questions (94.8% (109/115)). Second, the study was conducted between May and August. In Japan, the JCP scattering season is mainly from February to April and the mite breeding season, when allergen levels increase the most, is mainly from September to October. To avoid selection bias that could lead to the underestimation of SLIT, the study period was set to not coincide with the above two periods. In such a setting, memory distortions regarding symptoms may have affected caregivers’ responses. Third, the duration of SLIT in children was relatively short. This may have influenced caregivers' impressions of SLIT. Forth, caregivers of children who discontinued SLIT were excluded from the study. Although the reasons for treatment discontinuation may have varied, the survey results may have been different if these individuals had been included.

## Conclusions

In this study, we found that caregivers of children with allergic rhinitis who practiced SLIT felt more treatment satisfaction than burden for SLIT because the symptoms of allergic rhinitis in children were relieved or improved. Further statistical analysis showed that the treatment burden among caregivers was strongly associated with inappropriate adherence to SLIT in their children. Reducing the treatment burden among caregivers may not only improve adherence to SLIT in pediatric patients but may also provide a good prognosis for pediatric allergic rhinitis. We would like to conduct an intervention study with caregivers who feel burdened by SLIT, focusing on the outcome of removing the burden from the caregivers on pediatric SLIT adherence.

## Appendices

**Table 4:** Study questionnaire Credits: Sadayuki Nagai MD, PhD

Content	Answer
1. Date which you answered	Month ( ) / Day ( )
2. What is your relationship to your child? (e.g., mother, father, etc.)	( )
3. How old is your child?	( years old)
4. What is your child's gender?	(boy・ girl)
5. Which type of sublingual immunotherapy is your child receiving?	(Mitecure^®^, Cedarcure^®^, both tablets)
6. How and why did you start sublingual immunotherapy? (multiple-choice answers)	(Recommended by this hospital・recommended by other clinic・you or your family were practicing・your friends were practicing・interested on website or SNS・interested on magazines・other reasons)
7. What allergic symptoms prompted your child to start sublingual immunotherapy? (multiple-choice answers)	(Runny nose・sneezing・nasal congestion・nosebleed・itchy eyes・itchy skin・bronchial asthma・urticaria・other symptoms)
8. What did you expect from the treatment before starting sublingual immunotherapy? (choose one)	(Complete disappearance of symptoms ・ both symptoms and anti-allergy medication would decrease, making daily life easier・ not much hope, just a little relief of symptoms ・ other reasons)
9. Please give us your honest opinion about the sublingual immunotherapy currently being offered. First, please choose the most appropriate answer in parentheses regarding your impression of the treatment. Next, if possible, please feel free to write anything you want in the "satisfaction" and "burden" columns.	(I feel satisfied with the treatment・ I feel burdened by the treatment・neutral) Reasons why you say “I feel satisfied with the treatment” ( ) Reasons why you say “I feel burdened by the treatment” ( )
10. Would you recommend sublingual immunotherapy to your friends or family?	(Yes・no・neutral)
